# Accurate Human Tissue Characterization for Energy-Efficient Wireless On-Body Communications

**DOI:** 10.3390/s130607546

**Published:** 2013-06-10

**Authors:** Mónica Vallejo, Joaquín Recas, Pablo García del Valle, José L. Ayala

**Affiliations:** 1 Grupo de Automática de la UNAL, Departamento de Energía Eléctrica y Automática, Facultad de Minas, Universidad Nacional de Colombia - Sede Medellín, Cra 80 No.65-223, Medellín, Colombia; 2 Department of Computer Architecture and Automation, Complutense University of Madrid, C/Profesor José García Santesmases, s/n, Madrid 28040, Spain; E-Mails: jrecas@fdi.ucm.es (J.R.); pgarciav@fdi.ucm.es (P.G.V.); jayala@fdi.ucm.es (J.L.A.)

**Keywords:** Wireless Body Sensor Networks (WBSNs), human body communication, Received Signal Strength Indication (RSSI), body type, tissues, electromagnetic

## Abstract

The demand for Wireless Body Sensor Networks (WBSNs) is rapidly increasing due to the revolution in wearable systems demonstrated by the penetration of on-the-body sensors in hospitals, sports medicine and general health-care practices. In WBSN, the body acts as a communication channel for the propagation of electromagnetic (EM) waves, where losses are mainly due to absorption of power in the tissue. This paper shows the effects of the dielectric properties of biological tissues in the signal strength and, for the first time, relates these effects with the human body composition. After a careful analysis of results, this work proposes a reactive algorithm for power transmission to alleviate the effect of body movement and body type. This policy achieves up to 40.8% energy savings in a realistic scenario with no performance overhead.

## 1. Introduction

The increasing use of wireless networks and the constant miniaturization of electrical devices have empowered the development of Wireless Body Sensor Networks (WBSNs) as a special case of Wireless Sensor Networks (WSNs). In these networks, various sensors are attached on clothing, on the body, or even implanted under the skin. The wireless nature of the network and the wide variety of sensors offer numerous new, practical and innovative applications to improve health care and the Quality of Life, or monitor the performance of professional sportsmen. The sensors of a WBSN measure for example the heartbeat, the body temperature, or record a prolonged electrocardiogram [1].

In a common WBSN scenario, the user is fully functional and develops a physical activity with regular exercise and movement. The sensor nodes are strategically placed on the body according to the bio-signal to be captured. Usually, the MAC protocol used for the wireless communication is IEEE 802.15.4 [2], conceived for low-power, low-cost and low-speed communications.

The operation of the WBSN is determined, among others, by the proper communication between the wireless links. The wireless communication is very sensitive to several factors like the model and direction of the antenna, the distance, external disturbances (EM radiations in near spectral frequencies) and the presence of obstacles for the signal [3,4]. In WBSN, the body acts as a communication channel for the propagation of electromagnetic (EM) waves, where losses are mainly due to absorption of power in the tissue [5], which is dissipated as heat. The dielectric properties and penetration depth in the tissue in the frequency band of interest are strongly correlated with its water content [6]. Hence, depending on the type of tissue, if it is mainly composed of water, such as brain, muscle and skin, the EM-waves are attenuated considerably before they reach the receiver due to their high permittivity and loss [7,8]. However, it is important to consider that conductivity and permittivity values of the tissues vary for every individual because of many different factors such as age and anatomy [7,9], and additionally, the penetration depth decreases as the frequency increases. Therefore, according to several authors [7,8,10,11], at high frequencies like 2.45 GHz, the propagation takes place around the body surface, as combination of line of sight (LOS), multi-path and surface and creeping waves. However, these works lack a comprehensive study in a real environment where the specific composition of the body mass is responsible of the attenuation phenomena.

Very few attempts to characterize electromagnetic (EM) propagation around the human body have been made. This is probably because the properties defining EM propagation around the human body are complex, rendering the development of a simple mathematical model quite difficult. The human body has a complex shape consisting of different layers (e.g., tissues), each with its own permittivity and conductivity. Our goal is not to provide such an analytical model, but to perform an accurate experimental characterization of the channel, detecting the factors that alter the signal integrity, and to develop the contention mechanisms that save the wireless transmission energy while maintaining the quality of service.

On the other hand, the movement of the body plays an important role in the strength of the received signal. The positions adopted by the body, the body blocking the line of sight between the nodes, and the relative movement of arms and legs have proved to be responsible of up to 20 dB of attenuation [12]. Some authors have started to analyze this phenomenon [10,13–17]. However, these studies did not evaluate other variables related to the quality of the link, did not perform a methodical study and did not propose a contention mechanism to alleviate the negative impact of these factors.

The work presented in this paper addresses the following goals:

1. We show the effects of the dielectric properties of biological tissues in the signal strength.
2. We relate the effect of the biological tissues with the human body composition. Therefore, a relation of the body type (biotype) with the signal integrity is found.
3. We analyze the effect of simple and complex body movements in the quality of the received signal.
4. After a careful analysis of results, we propose a reactive algorithm for power transmission to alleviate the effect of body movement and body type. This policy tunes the transmitted power to reduce the energy consumption of the node and maintain the quality of service.

The rest of this paper is organized as follows. In Section 2 we review the related work in experimental characterization of on-body and off-body radio channels. In Section 3 we present the experimental setup, the executed tests and the obtained results. In Section 4, we analyze the effect of simple and complex movement of the body in the quality of the received signal and, also, we propose a reactive methodology to alleviate the negative effects of movement and body type, and to tune the transmitted power for reducing the energy consumption of the node. The final section summarizes the conclusions and discusses future works.

## 2. Related Work

In the literature, we can find several studies that analyze the interaction of the body with the electromagnetic signals. In [18], the authors present several studies that analyze the electromagnetic energy absorption in different human body models due to body-worn UWB antennas, at frequencies of 3, 6 and 8 GHz. With this study, the authors discover that, for very small distances between the body and the antenna (< λ/25), the increase of the unaveraged SAR (Specific Absorption Rate) due to the easier electric field penetration through the skin tissue is caused by a lower effective dielectric constant of the layered model. However, the impact of the layered composition of the human body becomes stronger for distances between the antenna and body bigger than λ/25. In this case, the increased SAR is caused by the standing wave effect.

Recently, there is an increasing interest on modelling the body composition for electromagnetic analysis. This is a strong requirement for the analysis of implantable antennas where the access to the device for characterization is almost impossible. Works like [19] present several body models, based on a single equivalent layer and based on a three layer structure, for the study of propagation losses of body implanted antennas at the ISM bands of 433 MHz, 915 MHz, 2,450 MHz and 5,800 MHz. The authors present as well the effects of the thickness of the different body tissue layers.

However, none of these works are related to our communication scenario of wireless body sensor networks, nor perform a complete experimental study under the realistic experimental conditions. However, their results help to explain the complex behavior observed in real experiments.

Several authors have examined the impact of human body on wireless communications at radio and microwave frequencies. In [11], the author evaluates, through simulation, the effects of tissue type on the polarization of radiation at 2.45 GHz by a dipole antenna, for different on-body channels. Simulations were done with three different tissue types: muscle, bone and fat of a male of 170 cm in height. Three important conclusions summarize the results shown in this paper: (1) the fat tissue has the highest average path gain and the muscle has the lowest; (2) for all the antenna combinations, the belt-chest channel provides the highest path gain while the belt-head and the belt-back channels yield the lowest path gain; (3) a vertically polarized antenna has omnidirectional radiation pattern and it is less affected by the surrounding environment, in comparison with a horizontally polarized antenna, which does not radiate equally in all directions.

On the other hand, the movement of the body plays an important role in the strength of the received signal. The positions adopted by the body, the body blocking the line of sight between the nodes, and the relative movement of arms and legs have been shown to be responsible of up to 20 dB of attenuation [12]. Some authors have started to analyze this phenomenon with Intel Mote 2 devices placed at the chest and ankle of the user, showing packet misses around 24%–28% [13]. Similar work has been done for sleeping users [14], which showed losses greater than 10%. However, these studies did not evaluate other variables related to the quality of the link, did not perform a methodical study and did not propose a contention mechanism.

The effect of the users in movement has also been studied by several authors in the literature. The movement of the antenna position impacts negatively on the attenuation of the received signal [10,15–17]. In [20,21], the authors evaluate a walking patient and the effect on the Received Signal Strength Indication (RSSI). Taparugssanagorn in [22], presents a series of UWB WBAN measurements in the frequency range of 3.1–10 GHz to compare two different types of antennas. The authors in this work also investigated the effects of body motion and concluded that, if ignored, the fluctuations of the radio channels under such dynamic situations can cause severe performance problems in system design.

However, none of these works evaluated complex but common natural movements, estimated the effect of body type, or proposed a mechanism to alleviate the negative impact of these factors.

## 3. Experimental Tests and Results

The work presented in this paper in the field of WBSNs has been performed considering different communication scenarios: communications near biological tissue and on-body propagations. For the case of communications near biological tissue, we have investigated the effects caused by the different types of tissues when they interrupt the visibility of the antennas. Our purpose is to differentiate the contribution of every type of tissue in the attenuation and absorption of the energy transmitted. In addition, we aim to understand the wave propagation phenomenon on flat regions of a biological body (e.g., communication from the front to the back) when the tissue layer is either thin or thick. For the on-body case, we investigated the effect of body positions and human movement in the quality of the wireless communication performed in an indoor environment. In these experiments, the antennas were placed directly on the subject’s clothes. This section presents the experimental setup and the measurement results for three different scenarios:

- **Communications near biological tissue in an anechoic chamber:** we have investigated the impact of biological tissues in the signal strength. The measurements were done at various distances throughout the Line Of Sight (LOS) and through the None Line Of Sight (NLOS) in the cases that the tissues interrupt between the antennas. Different porcine tissues were used as a replacement of human body parts and they were arranged as a layered body.
- **Communications near biological tissue in an outdoor environment:** this scenario presents the same experimental conditions as the first one. In addition, new measurements were taken for varying numbers of layers (from 1-layer to 3-layers) in order to evaluate the effects of the thickness of the biological body.
- **On-Body communication channels:** we have investigated the temporal variations in the quality of two communication links. This characterization was performed with human subjects placed at fixed positions and performing realistic movements in an indoor environment.

### 3.1. Experimental Setup

Two sensor nodes have been used in our experiments, the Shimmer [23] and Samsi [24]. The Shimmer node is equipped with an ultra-low-power 16-bit microcontroller (TI MSP430) that runs at a maximum clock frequency of 8 MHz and includes 10 KB of RAM and 48 KB of Flash. This platform has also two radio links, IEEE 802.15.4-compliant CC2420 transceiver [25] and Bluetooth radio (the last one has not been used in our experiments because of its power consumption). The transceiver CC2420 has sensitivity threshold of −94 dBm and provides eight different programmable power transmission levels, from the maximum level at 0 dBm and a current consumption of 17.4 mA, to the minimum level at −25 dBm and a current consumption of 8.5 mA (see Table 1). This sensor node also uses a GigaAnt Rufa SMD antenna operating at 2.4 GHz with omnidirectional radiation pattern. From the software viewpoint, we have ported FreeRTOS [26] for this platform, which is a portable, open source, hard real-time mini kernel that includes support for the microcontroller and the IEEE 802.15.4-compliant radio chip used by Shimmer.

The Samsi node developed by ARTICA [27,28] is equipped with an ultra-low-power 16-bit microcontroller (TI MSP430) that runs up to 25 MHz and includes 16 KB of RAM and 256 KB of Flash [28]. This platform has also an ATmega128RFA1, which is an IEEE 802.15.4 compliant single-chip that integrates an 8-bit microcontroller running at 16 MHz. It also includes 128 KB flash memory, 16 KB RAM and a transceiver AT86RF231. This radio chip has a receiver sensitivity of −100 dBm and a programmable output power ranging from +3.5 dBm and current consumption of 14.5 mA to −16.5 dBm and current consumption of 8 mA (see Table 1) [29]. This sensor node also uses a 2.4 GHz ceramic chip antenna of Johanson technology, which exhibits linear polarization and provides a near omnidirectional radiation pattern. From the software viewpoint, this platform uses FreeRTOS as operating system, and the MACv2.6.1 stack library from Atmel.

**Figure 1.**
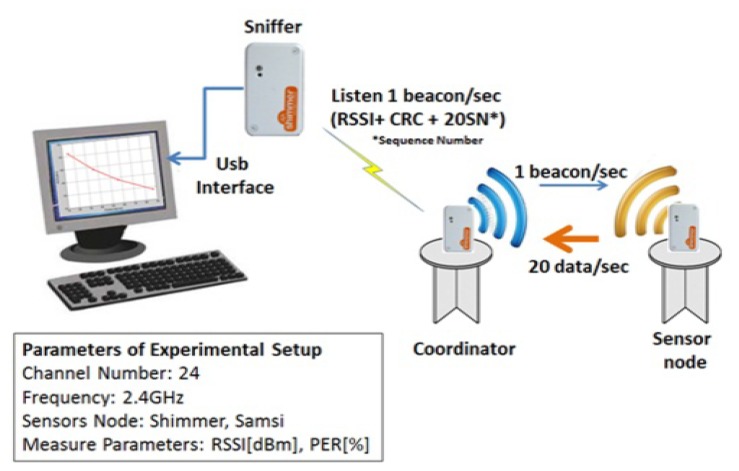
Experimental Setup.

The nodes were tuned to 802.15.4 channel number 24, in star topology. The coordinator sent beacons packets of 40 bytes of payload, at a fixed rate of 1 packet per second at the maximum power level. The other node answered to beacon with data packets of 30 bytes of payload, at a fixed rate of 20 packets per second. The coordinator of the system is programmed to provide RSSI reading, CRC bit reading, and the sequence number for every data packet received from the node sensors at different power transmission levels. Then, the coordinator integrates and sends this information in the beacon packet payload. An external node passively listens to the beacon packets and takes periodic noise floor measurements. This node is connected to PC through the USB interface. A Java interface runs in the PC and draws in real time the respective RSSI reading of each packet and the RSSI noise measure during the tests. The RSSI level is recorded over of 10 seconds for each measurement (see Figure 1).

The two experimental scenarios described at the beginning of Section 3 were executed as follows. Initially, the experiments were performed using the Shimmer node. These initial experiments were conducted in an anechoic chamber to have a controlled environment free of unwanted reflections and ensure that the effects were caused only by the presence of biological material. Once these measurements were collected, the experimental work followed in the outdoor environment with the purpose of comparing and validating the results with those taken in the anechoic chamber. After that validation, the rest of the experiments could be continued in such environment. The experimental work concluded that the outdoor environment was appropriate to continue performing the experiments as the results obtained in both environments were very similar. Finally, we completed the experimental work in the outdoor environment with a new sensor node in order to verify that the received signal power behavior in the presence of biological tissues and/or persons is independent of radio and antenna technology of the node and therefore the proposed optimization policy could be extended to any node. In the following sections, we will present the results obtained with this methodology and the analysis of results.

### 3.2. Communications near Biological Tissue in an Anechoic Chamber

The experiments were conducted in the anechoic chamber facility in Technical University of Madrid (UPM) using the experimental setup aforementioned. The anechoic chamber has dimensions 7.3 m × 4.3 m × 4.3 m, being suited for the measurements in frequency range of 1.5 to 20 GHz [30]. The anechoic chamber was used to measure the impact of the biological tissues on RSSI in a controlled environment with minimum unwanted reflections

In this experiment, we distinguished two situations for a pair of sensor nodes (Shimmer): LOS (Line-of-Sight) and NLOS (Non-Line-of-Sight) due to biological tissue. The tissues were placed in the middle point between the transmitter node and the receiver node, and at the same height of the devices. The transmitter and receiver nodes have their antennas with identical orientations, *i.e.*, placing the nodes parallel in a straight line, facing one another and in the same plane to provide the best communication range. The onboard SMD antenna demonstrates an omnidirectional radiation pattern according to datasheet. Porcine tissues have been used because of their easy accessibility and because their dielectric properties and biological responses are similar to human tissues [31,32]. Table 2 shows the relative permittivity (*ε*) and the conductivity (*σ*) of several types of human and porcine tissues, including skin, fat and muscle at the frequency of 2.45 GHz [11,33–35]. As can be seen, fat tissue has a significantly lower permittivity and conductivity with respect to other tissues.

In order to characterize the attenuation of the biological tissues, the biological material was defined as consisting of three and four planar layers. For the first case, we used three pork fat slices of 1 cm in thickness for modeling a body tissue of 3 cm in thickness; for the second case, the four-layered body is composed of a pork skin slice of 0.5 cm, a pork fat slice of 1 cm, a pork loin slice of 1 cm, and a backbone piece. The Shimmer sensor nodes were placed on supports at a height of 25 cm above the ground and the measurements were done at the minimum power transmission level of −25 dBm, and for distances between the sensor nodes of 15, 36, 57, 87 cm.

**Figure 2.**
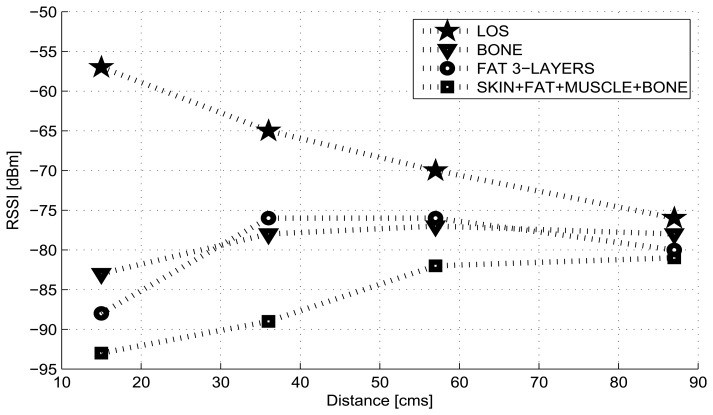
RSSI *vs.* Distance for LOS/NLOS links in anechoic chamber.

From results shown in the Figure 2, we can observe an attenuation between 20 dBm to 30 dBm for the NLOS cases due to pork tissues in relation to free space. In the NLOS situations, the received power is greatly reduced for the four-layered tissues case that includes skin and muscle (pork loin) compared with the three layers of pork fat. This behavior can be explained by the fact that muscles and skin have higher water content and therefore higher permittivity, higher conductivity and higher loss [8,36]. Furthermore, the received power, for the cases of pork fat and pork bone, shows close values because both types of tissues have similar dielectric properties [8].

From Figure 2 it is possible to observe that NLOS curves do not show a linear-logarithmic characteristic as the LOS curve; instead, a large attenuation is experienced when the nodes are in the position closest to the tissues, and then it changes slightly as the distance between nodes extends. The reason is that when the area of the tissues that interrupts the transmission channel becomes small relative to the distance between the nodes, the diffracted signal becomes stronger [37].

### 3.3. Communications near Biological Tissue in an Outdoor Environment

**Figure 3.**
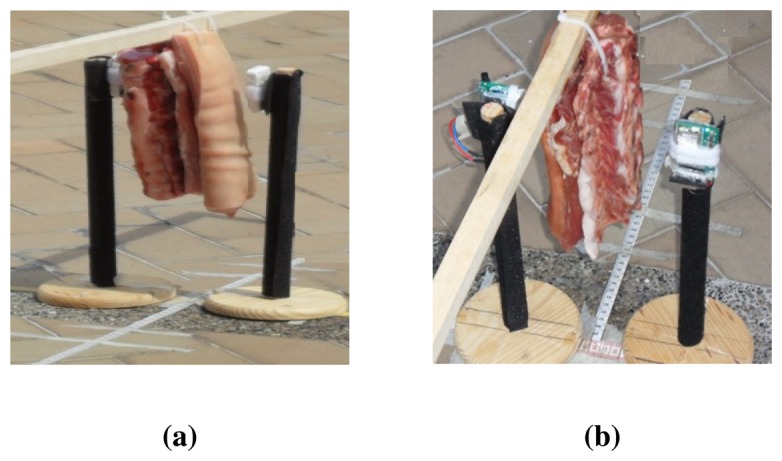
NLOS due to four-layered porcine tissue in outdoor environment. (**a**) Shimmer nodes; (**b**) Samsi nodes.

The measurements were done using the Shimmer and the Samsi nodes in outdoor environment, as it is shown in Figure 3. The chosen site is a plain open field apart from any vehicle or person path. It is considered a reflection-free environment because it helps eliminate attenuation due to the diffraction caused by the floor and the constructive and destructive interference due to multipath. Also, as recommended by the author in [38], the sensor nodes were placed in a place with no obstacles in a radius of 2λ meters (0.25 mts). The experiment was performed for all power transmission levels of both nodes; there are 8 levels for the Shimmer and 16 levels for the Samsi. This experiment has been conducted under the same conditions used for the anechoic chamber.

#### 3.3.1. Effects of Dielectric Properties of Biological Tissues on the Signal Strength

**Figure 4.**
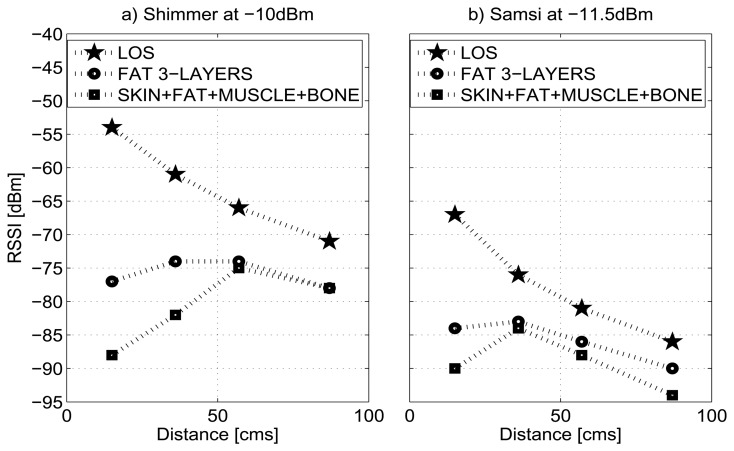
RSSI *vs.* Distance for LOS/NLOS links in outdoor environment.

The measurements collected in the outdoor environment with the Shimmer nodes were compared and validated with those taken in anechoic chamber; after that, we continued making the remainder of the experiments in outdoor environment as both results sets are very similar. Figure 4 plots the RSSI values for the 4-layered tissue (comprising skin, fat, muscle and bone) and for the 3-layered fat tissue, at −10 dBm for the Shimmer node and at −11.5 dBm for the Samsi node. As shown the figure, the RSSI exhibits a similar trend for both nodes in relation to the measures obtained in the anechoic chamber.

According to the results, the heterogeneous material shows greater attenuation than homogeneous material. This is because on the heterogeneous structure of biological tissues, the electromagnetic field distribution is complex and depends, among others, on the dielectric properties of each tissue and on the presence of the different interfaces, (e.g., air/tissue and tissue/tissue) [39]. In fact, the relative permittivity, the conductivity and frequency determine the reflected and transmitted energy of the EM wave through interfaces between different tissues. In each layer boundary, the field is partly reflected and transmitted, which causes the superposition of wave fronts and, finally, the standing wave in these regions.

Comparing the dielectric properties of the tissues (Table 2), we noted that the skin and muscle have very similar properties; however, skin becomes the most reflective layer. Thus, most of the power in the incident wave is dissipated in the skin layer and penetrates relatively little to deeper layers of tissue.

From the point of view of the node, the Samsi node shows a higher attenuation for the LOS case, and its effect on the signal strength is more noticeable for the heterogeneous layered medium compared with the Shimmer node.

#### 3.3.2. Effects of Homogeneous and Layered Biological Tissues on the Signal Strength

**Figure 5.**
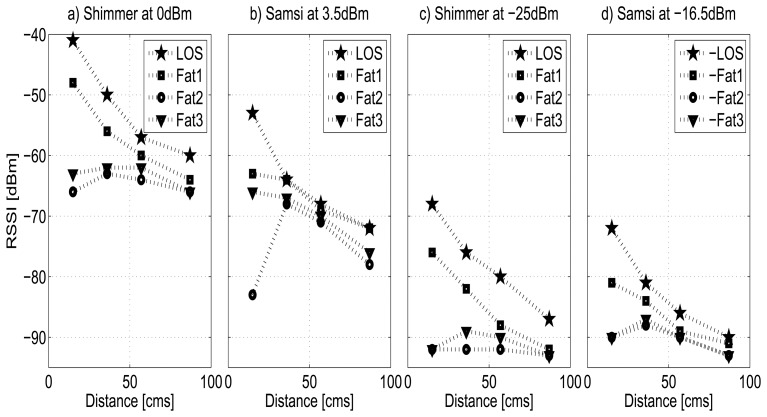
Homogeneous and layered biological tissue: fat tissues.

Figure 5 shows the measurements for the homogeneous and the layered fat tissue as function of the distance between the nodes. In the figure, we denote Fat1 as a fat tissue slice of 1 cm and Fat2 and Fat3 as layered tissues of two and three slices of 1 cm respectively. Although in the figure only the results for the Shimmer node at 0 dBm and −25 dBm and for Samsi node at 3.5 dBm and −11.5 dBm are shown, the experiments were done for all power transmission levels of both nodes and it was verified that the results exhibit the same behavior for all cases.

From the figure, it is clear that, at a fixed distance, the introduction of the layered tissue (Fat2 and Fat3) causes the signal strength to decrease significantly compared with the homogeneous tissue (Fat1). This is particularly true when the nodes are very near to the tissue, where signal strength can drop up to 20 dBm. However, we also observed that the influence of the number of layers (thickness) of the layered medium does not provide significant differences. In contrast, it seems that the influence of a medium’s thickness decreases as the medium becomes thicker (Fat3). According to [40], this is because at 2.4 GHz, the layered medium is then increasingly acting as a homogeneous medium.

From the viewpoint of the transmission power, it is observed that the strength of the received signal decreases accordingly to the lowest transmission levels, both for homogeneous and layered tissues, from 29 to 24 dBm, and for the Shimmer and the Samsi nodes respectively. Despite this, the RSSI values are always above the sensitivity threshold, and the transmission is always performed with an average packet loss lower than 10%. This is because the layers of fat tissue used are much thinner than the corresponding penetration depths (113 mm at 2.45 GHz) [7] and once the electric field crosses the fat-fat boundary, it attenuates monotonously. Thus, because the homogeneous tissue (Fat1) is rather transparent for radio waves, it exhibits similar behavior to the LOS curve.

By comparing the sensitivity of the radios of both nodes, we found that although the Samsi node provides power transmission levels higher (+3.5 dBm) than the Shimmer node (0 dBm), the first one also shows higher attenuation for all tissues in relation with the Shimmer node. This fact can be explained by the characteristics of the radio and the antenna integrated in the device.

**Figure 6.**
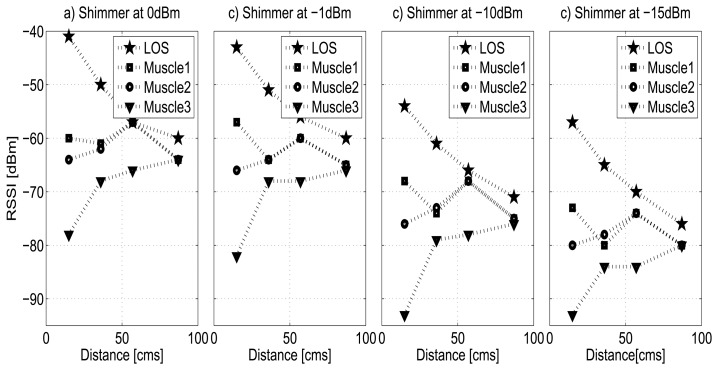
Homogeneous and layered biological tissue: muscle tissues.

Figure 6 shows the measures for the homogeneous and layered medium of the muscle tissue (pork loin) as function of the distance. Again, the experiments were done for all power transmission levels and it was verified that the results exhibit the same behavior for all cases on both nodes. In order to analyze in detail the relation between the signal strength and the variation of the power transmission, in the figure only the results for the Shimmer node are shown at four different power transmission levels: 0, −1, −10 and −15 dBm. In the figure, we denote Muscle1 as a pork loin tissue slice of 1 cm and Muscle2 and Muscle3 as layered tissues of two and three slices of 1 cm each.

In relation to the behavior of homogeneous and layered medium using muscle tissue, it is clear that muscle3 causes a significant drop on the signal strength, especially when the nodes are very close to the tissue where the attenuation can be up to 37 dBm with respect to the LOS curve at 0 dBm. On the other hand, except for the point closest to the tissue, it seems that the thickness of muscle 2 is neglected by the EM wave and its effect on the signal strength is similar to that shown by muscle 1. Interestingly, every curve follows a monotonic pattern as we have observed for other tissues; a similar phenomenon has been also reported in [37,41,42] where the authors attribute the fact to diffraction suffered by signal when the area of the tissues becomes small relative to the distance between the antennas.

In the figure, it can be observed that the strength of the received signal decreases according to the use of the lowest power levels, as we mentioned previously. For the curves of LOS and muscle 1, and 2, in the power levels from 0 dBm to −15 dBm, the difference in the attenuation of the signal is less than 6 dBm. However, the muscle 3 is considered a critical case for the lower power levels (from −10 to −25 dBm), where the RSSI value to the closest distance of the tissue falls dangerously until overtaking the sensitivity threshold of the radio (−94 dBm).

#### 3.3.3. Effects of Human Body Characteristics on the Signal Strength

In this section, our goal is to observe the effects of the different human body types on the RF wave propagation when they interrupt the direct path between a transmitter and receiver. We present a case of study with the Shimmer node at −10 dBm for four subjects (4 males) holding a variety of body types, whose characteristics are presented in the Table 3. Subject 1 and Subject 2 are taller than the average, close to 190 cm, but have different body masses. While subject 1 has a Body Mass Index (BMI) equal to *BMI*_1_ = 28.16, which corresponds to overweight, subject 2 has *BMI*_2_ = 23.70, which is in normal range. Subject 3 and 4 have average height with *BMI*_3_ = 24.54 and *BMI*_4_ = 27.68, which correspond to average and overweight. For every subject, his body was placed for the experiments in the middle point between the transmitter node and receiver node, which were located parallel in a straight line, facing one another and in the same plane. The measures were done for various parts of the body (chest and belly) blocking the line-of-sight (LoS) at various distances. Each subject was standing still for a period of 30 s. After this initial setup, we followed the same experimental procedure as explained in previous subsection.

Although the measurement campaign with biological tissues helped us to understand the signal strength variability in the presence of lossy mediums, characterized by different values of the relative permittivity and the conductivity, the behavior observed for the porcine tissues cannot be extended to explain the RF behavior caused by actual human body. The human body has a complex shape and consists of different layers, tissues and organs, each with its unique dielectric properties.

**Figure 7.**
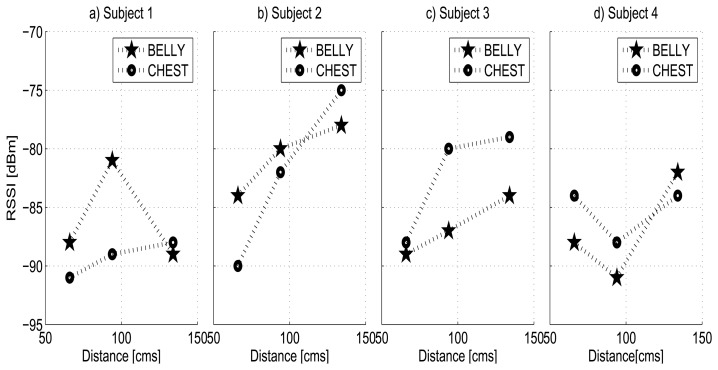
RSSI measures of four subjects with Shimmer node at −10 dBm.

Figure 7 shows the RSSI measures of the chest and belly for all subjects. From the figure we can observe the different patterns in RF wave propagation for each one of them. Therefore, e.g., for Subjects 1 and 2, the chest shows more attenuation than the belly, while for Subjects 3 and 4 the opposite effect occurs. These facts can be explained because the human body is an inhomogeneous medium (it consists of different types of tissues, organs and liquids) of complex structure with large anatomical differences between subjects, such as size, compositions and thickness.

On the other hand, the measures show the prevalent effects of the reflections and of the diffracted signal from the body parts of the subjects. A stronger attenuation for the closest point to the human body, followed by slight changes in the attenuation value when the distance extends, is the consequence not only of the dielectric properties but also of the shape (curvature) and the dimensions of individual body parts. According to [43], if the direct path between the transmitter and receiver is obstructed by an obstacle, waves can travel into the shadow zone behind the obstacle.

Another noticeable aspect that can be observed in Figure 7 is that the measured RSSI values are below −75 dBm for all subjects and, although the signal does not fall below the sensitivity threshold, we can expect a dramatic increase of packet loss and consequently link loss for lower power levels, specially for the Subject 4.

### 3.4. On-Body Communication Channels

**Figure 8.**
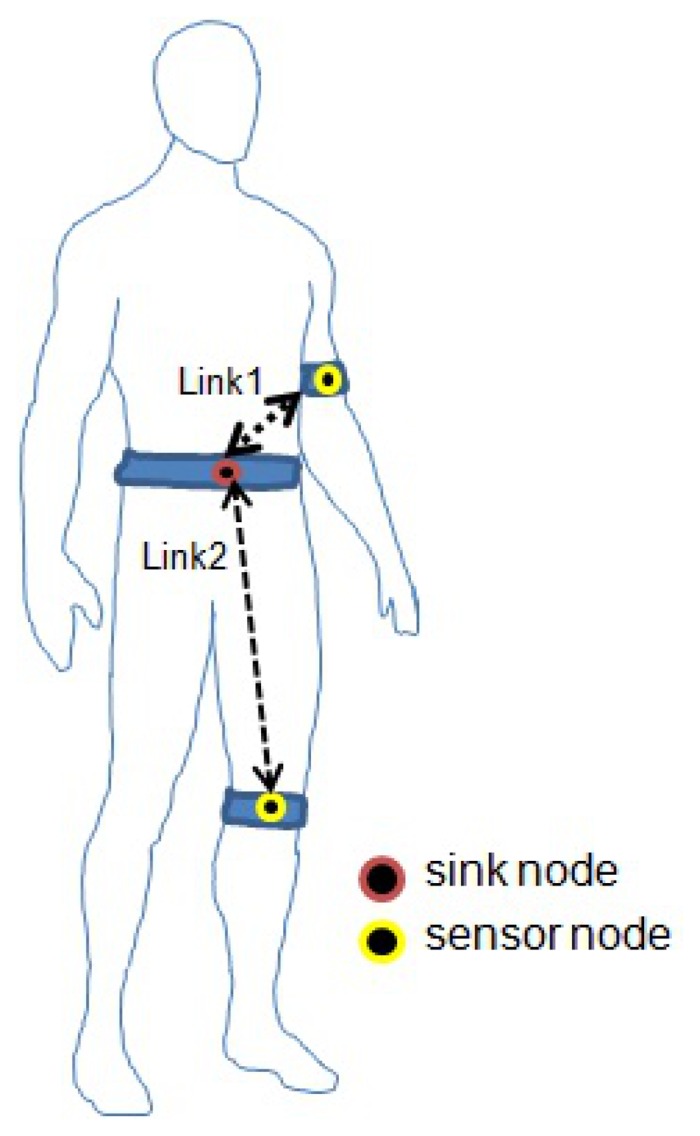
Node Position.

The goal of this experimental scenario is to analyze the effect of simple and complex body movements in the quality of the received signal; for this, we considered the same subjects described in the previous Subsection 3.3.3. The nodes were placed on the subject’s body following a star topology, with the coordinator placed in the waist (just over the navel), and the node sensors in the left arm (link L1) and in the left knee (link L2), as shown in Figure 8. For each subject and scenario, the measurements were repeated at four power transmission levels for each sensor node. For the Shimmer nodes, we tested at 0 dbm, −7 dBm, −15 dBm and −25 dBm; for Samsi nodes we tested at 3.5 dBm, 0.5 dBm, −6.5 dBm, and −16.5 dBm. All the results were obtained in controlled conditions to minimize the effect of interfering EM-waves (WiFi, 3G, solar radiation, *etc*).

We planned four experimental scenarios to investigate the temporal variations in the quality of the two links due to stationary and dynamic positions:

- **Scenario 1:** the subject sat on a chair performed five movements of the arms (for Link 1): (1) hands on thighs, this link and position will be denoted as L1/P1; (2) arms crossed, L1/P2; (3) arms extended forward, L1/P3; (4) arms extended up, L1/P4; and (5) arms extended to both sides, L1/P5.
- **Scenario 2:** the subject sat on a chair performed four movements of the legs (Link 2): (1) leg in 90° angle with the body, L2/P1; (2) left leg crossed over the right knee, L2/P2; (3) right leg crossed over left knee, L2/P3; and (4) leg extended forward, L2/P4.
- **Scenario 3:** the subject walked (for L1 and L2).
- **Scenario 4:** the subject performed a complete sequence (for L1 and L2): (1) sat with hands on thighs; (2) stood with arms parallel to the body; (3) walked 10 steps; (4) suspended, and extended the arms up. Each step is 5 seconds long.

According to the experimental results, we can approach the analysis for three remarkable variables: threshold, lossless positions and body types. For the first, we show that the results for both types of sensor nodes (Shimmer and Samsi) are very similar; therefore, for the other variables, only the results of the Shimmer are shown.

#### Threshold

**Figure 9.**
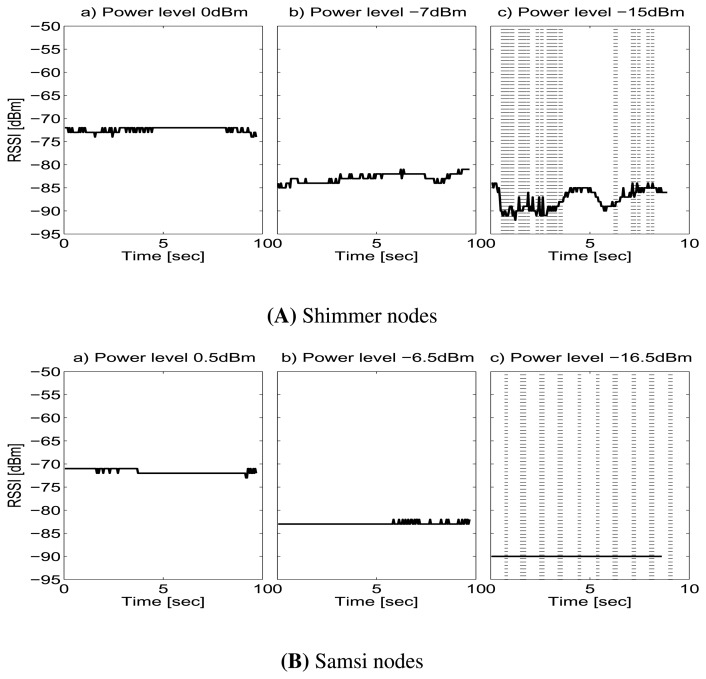
Power variations for Subject 1 L1/P2.

The threshold effect can be observed in Figure 9. The background noise presents a constant value of −98 dBm and does not affect the radio link. The figure shows the RSSI and the Packet Error Rate (PER) (dotted line) for the subject 1, link L1, and position P2 at three different power transmission levels for each node, Shimmer and Samsi nodes. As can be observed, the RSSI values are far from the threshold for both kind of nodes, in Figure 9(A-a,b,B), therefore, there is no loss of packets. In the Figure 9(A-c,B-c), the energy received comes closer to the sensitivity threshold and more packets do not reach the destination. Hence, for RSSI values below −85 dBm for Shimmer nodes, and below −90 dBm for Samsi nodes, the packet loss increases dramatically, because the link quality varies radically near the sensitivity threshold [20]. Therefore, for specific positions, the RSSI and PER value can vary depending on the power transmission level. In this way, at higher power transmission levels, PER is minimal and RSSI is above of the threshold, but for lower power transmission levels, PER increases and RSSI may drop below the threshold.

#### Lossless positions

**Figure 10.**
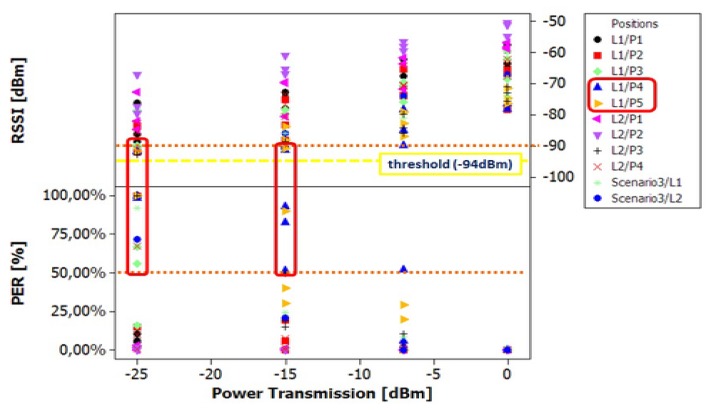
One-way ANOVA: RSSI, PER *vs.* Power Transmission.

For the purpose of analysis, we extracted statistical parameters like mean, standard deviation, minimum and maximum value of the RSSI and PER. The analysis of variance (one-way ANOVA) [44] was used as a statistical method as it allowed to determine whether the groups (positions, power transmission levels) are actually different in a single measured characteristic. From Figure 10 we can observe that L2 in positions P1–P2 are the most favorable positions because there is no packet loss for any subject, independently of the power transmission level used. This fact can be explained because the sensor nodes have direct line of sight in these configurations and are located at a short distance. So, these wireless links can be considered lossless.

For L1 in positions P1–P2 and L2 in positions P3–P4 we began to observe a mean packet lost of 12%. It can be explained due to shadows caused by some parts of human body, which reduces the LOS factor contribution to the received signal. The most critical positions in this group are L1/P2 and L2/P3 because the shadow area is deeper as compared with the other two positions. This fact has a greater impact when lower power transmission levels are used.

Finally, for L1 in positions P4 and P5 (arms extended up and arms extended to both sides), we can observe a large percentage of packet losses with PER values up to 50%, and for RSSI, a drop to below −90 dBm at power transmission levels from −15 dBm to −25 dBm. This can be explained due to the small scale fading caused by movements of a human and because the signal has to go through a wide body section and suffers more attenuation.

#### Body types

**Figure 11.**
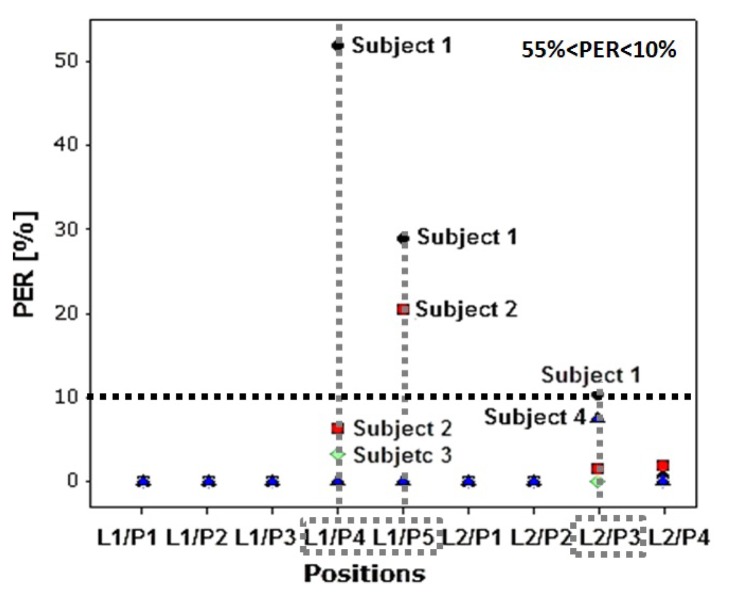
PER *vs.* Positions at −7 dBm.

To get the results, the radio was configured to transmit at −7 dBm and the measurements were made on steady periods of 10 seconds. Figure 11 and Table 4 show the Packet Error Rate (PER) for each subject in the different scenarios and links obtained with the Shimmer nodes.

We observed that for L1/P4 and L1/P5 previously identified as lossy positions, the Subjects 1 and 2 (both taller than the average) exhibit greater percentage of packet losses than Subjects 3 and 4. From these data we can conclude that longer arms affect the communications; if we move the sensor away from the waist, we can reach the point at which the RSSI is below the threshold. It is also noticeable that, while PER for L1/P5 is similar for Subjects 1 and 2 (about 25%), the L1/P4 PER is very different, 52% for S1 and 6% for S2. This is closely related to the body type: in P5 the blocking generated by the body is very similar (just the arm) and its circumference is very similar. However, in P4 we have a noticeable body blocking, the signal has to go through a big body section and suffers more attenuation for S1 (overweight) than S2 (average). The same effect can be found in L2/P3 for Subjects 2 and 3 compared with Subjects 1 and 4, *i.e.*, the higher the body mass, the stronger the body blocking.

## 4. Case Study

Common wireless sensor nodes, like Shimmer, Samsi, Mica Motes, TMoteSky, *etc.*, are equipped with low power 3-axis accelerometer sensors. The 3-axis accelerometers are able to measure the acceleration along the X, Y and Z axes, from which velocity and displacement can be estimated. It is also possible to detect body-position and posture as they are able to measure the gravity force and therefore can be used as an inclinometer [45].

In the following section, we devise a reactive algorithm for power transmission to alleviate the effect of body movement and body type based on the RSSI variation study presented in Section 3.4. This policy tunes the transmitted power to reduce the energy consumption of the node while maintaining the quality of service. The policy will be applied to both types of wireless sensor nodes studied in the previous sections, *i.e.*, Shimmer and Samsi.

### 4.1. Optimization Policy

Once the communication link is correctly characterized for each subject and scenario at different transmitted power, it is possible to adjust the transmission power using the movement detection based on accelerometry with low-complexity and low overhead. In order to reduce the energy consumption of the radio link, we will choose dynamically the lowest energy transmission level that requires the overall lowest energy budget following Algorithm 1.



**Algorithm 1** Optimization policy for a characterized radio link.
1:**procedure** Energy-Aware Send Data Packet(data)2: *accXY Z* ← *measureAcceleration*()3: **if**
*module*(*accXY Z*) >> 9.8 **then**4:  *radioPower* ← *MAXIMUM*5: **else**6:  *radioPower* ← *getOptimumLevel*(*accXY Z*)7: **end if**8: *status* ← *sendData*(*data, radioPower*)9: **while***status* <> *ACK*
**do**10:  *radioPower* ← *getMinimumLooselessLevel*(*accXY Z*)11:  *status* ← *sendData*(*data, MAXIMUM*)12: **end while**13:**end procedure**


The devised policy for lost packets works as follows: first we estimate if the node is moving using the module of the acceleration vector. If the value of the acceleration is bigger than |*g⃗*| (9.8*m*/*s*^2^), the node in motion that can be identified with **Scenario 3,** and as can be seen in Figure 10, and the radio power should be set to maximum in order to avoid packet losses (0 dBm and 3.5 dBm for Shimmer and Samsi respectively). Otherwise, if the acceleration is close to |*g⃗*|, the node is still and we can easily estimate the relative position of the node (the location on the body is fixed and known) through its orientation relative to *g⃗* vector. With the orientation and using the characterization data of Table 4, the optimum power level for radio transmission can be obtained. The experimental setup of accelerometers follows the ideas proposed in [45].

Once we transmit at optimum power level, the reception of the *ACK* has to be verified. If a packet does not reach the destination, this packet is re-sent at the minimum power level that has a packet delivery ratio above 99%, and therefore it is very unlikely that the packet must be re-sent again. As seen in Figure 10, this minimum power level considered *Lossless Level* in Algorithm 1 depends on the relative position of the nodes and the body type. This technique has a moderate energy penalty for retransmissions and can be considered as a good policy if we want to avoid saturation in the channel and packet queuing. Wireless Sensor Nodes usually have scarce amount of RAM memory, as seen in Section 3.1, and hence queue packets may lead to buffer overflows in the nodes.

**Figure 12.**
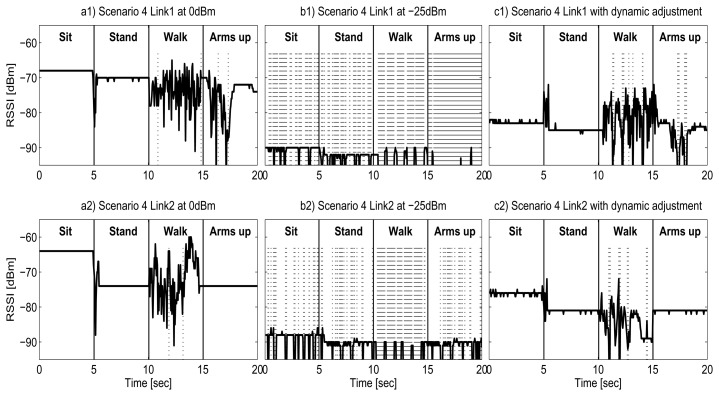
RSSI evolution for Shimmer in Scenario 4.

Figure 12 represents the change of RSSI for the Shimmer in the **Scenario 4** introduced in Section 3.4, for three different configurations: transceiver configured to transmit at *Maximum Power*, 0 dBm (Figure 12(a1,a2); at *Minimum Power*, −25 dBm (Figure 12(b1,b2)); and at *Optimum Power*, which corresponds with the proposed dynamic adjustment for energy savings (Figure 12(c1,c2)). This covers all possible energy levels available for the transceiver. At *Maximum Power* the RSSI has a mean value of around −70 dBm, much higher than the sensitivity of the transceiver and therefore the data transmission is lossless. At *Maximum Power* (0 dBm), the radio transceiver consumes an overall of 46.2 J. If we transmit at *Minimum Power*, the RSSI is very close to the sensitivity level (−94 bBm) and hence many packets are lost. The total energy consumption is 48.7 J, higher than the *Maximum Power* scenario because there are nearly 95% of retransmissions. In this situation almost all packets are sent twice: first at *Minimum Power*, with a high probability of not receiving an ACK, and then at *Lossless Level*, which corresponds to the *Maximum Power* in most of the cases for the Shimmer node.

Thanks to the dynamic policy in *Optimum Power* scenario, the transmission power is set to the optimum for every phase of the complex movement. The RSSI in Figure 12(c1) has a mean value of −82 dBm, always above −90 dBm but far form −70 dBm, and a limited number of retransmissions are required. With this configuration, we consume 29.9 J for transmission, which is translated into a reduction of 35.3% of the total energy in comparison with the maximum transmission power mode.

It should be noticed that in Figure 12(c2) we observe that the RSSI measured in the *Optimum Power* scenario during the “Sit” phase is far from the sensitivity threshold. It can be reasonable to think that the transmitted power might be reduced even more but, as the transmitted power has limited levels, reducing one level may incur a greater packet loss ratio that leads to an energy penalty due to retransmissions. For other transceivers with more power levels for transmission, the energy saving may be increased.

**Figure 13.**
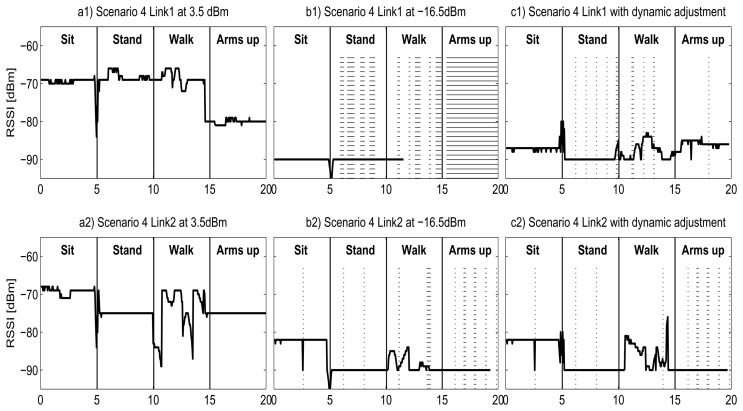
RSSI evolution for Samsi in Scenario 4.

Figure 13 presents the change of RSSI in the **Scenario 4,** in the same way as Figure 12, but for the Samsi nodes. It can be noticed that the behavior is similar, but with some differences. For the *Maximum Power* scenario (subfigure a), as in the Shimmer case, there is no packet loss. For the Samsi, this scenario corresponds to 3.5 bBm of transmitted power. In this situation, the RSSI is also much higher than the sensitivity of the transceiver, and corresponds to a total energy consumption of 38.3 J.

At *Minimum Power*, Figure 13(b), we observe a large packet loss ratio, but lower than *Minimum Power* level for the Shimmer. This is because even though the RSSI value is also close to −90 bBm, the sensitivity of the Samsi transceiver is higher (−100 dBm for Samsi *versus* −94 bBm for Shimmer), and therefore fewer packets are lost. The *Minimum Power* scenario has a total consumption of 26.5 J and, unlike the Shimmer, it is lower than the *Maximum Power*. This can be explained by two factors: first, the Samsi node has reduced packet loss ratio at *Minimum Power*, and second, the retransmissions have less energy penalty. In contrast to the Shimmer node, the Samsi exhibits a wide range of transmitted power levels, and thus is possible to find a *Lossless Level* with a smaller consumption than the *Minimum Power*.

Finally, the *Optimum Power* exhibits a RSSI very similar to the *Minimum Power* in most of the cases, and therefore its energy consumption is very similar, with a total of 22.7 J. This value corresponds to a reduction of 40.8% in comparison with the *Maximum Power* scenario, which is, as expected, a greater energy saving than the 35.3% obtained by the Shimmer.

Figures 12 and 13 also highlight other differences between nodes. During the walking period, the RSSI has great variations for the Shimmer during the entire phase for both L1 and L2 links, with periods very close to the sensitivity threshold, while the Samsi has less variations and is always far from the sensitivity thresholds, hence less packets are retransmitted, reducing the energy consumption in that period.

According to these results, the use of a fixed transmission power either wastes energy or hinders reliability; therefore, we must highlight that the presented reactive optimization policy is able to reduce the energy consumption due to data transmissions while maintaining the quality of the link for various wireless sensor nodes, different node locations and body types, provided a careful characterization is performed. We also expect to obtain increased energy savings for transceivers with wider transmitted power range. It can be noticed that this policy could be used jointly with the activation of power-saving modes (like sleep transition mode) to improve the expected energy optimization. However, we must consider that these low-power modes include a power and performance impact on the transition to the active state, which is only compensated when the low-power state lasts for a long time. In our dynamic scenario, the frequent change in the positions of the body, and therefore in the blocking of the line-of-sight, does prevent from using such low-power modes. As a future work, we could extend our transmission power control policy with node sleep scheduling strategies in order to cover other scenarios where the movement of the subject is not so frequent.

## 5. Conclusions

This work has presented an accurate characterization of the communication channel for on-body wireless networks. The experimental work included in this paper has detected the parameters that impact on the quality of the radio link and the received signal: fundamentally, the tissue characteristics (muscle, fat and bones), the human body composition and structure (body type), and the blocking of the line-of-sight that appears during the natural movement. For the first time, an accurate characterization of this radio channel has been performed in a realistic scenario with different subjects that exhibit the required variability in their body composition.

The obtained experimental results and the analysis of them have driven the implementation of a reactive management policy that, by tuning the power transmission levels, is capable of reducing the energy consumption of the radio chip to a minimum while maintaining the quality of service.

## Figures and Tables

**Table 1.** Output power settings and typical current consumption.

**Shimmer Node**		**Samsi Node**			

**Tx Power [dBm]**	**I [mA]**	**Tx Power [dBm]**	**I [mA]**	**Tx Power [dBm]**	**I [mA]**
0	17.4	3.5	14.5	−1.5	9.13
−1	16.5	3.3	13.9	−2.5	9
−3	15.2	2.8	12.4	−3.5	8.88
−5	13.9	2.3	11.2	−4.5	8.76
−7	12.5	1.8	10.3	−6.5	8.55
−10	11.2	1.2	9.86	−8.5	8.36
−15	9.9	0.5	9.59	−11.5	8.15
−25	8.5	−0.5	9.31	−16.5	8

**Table 2.** Electrical properties of human and porcine tissues at 2.4 GHz.

**Tissues**	**Relative Permittivity (***ε***)**	**Conductivity ((***σ***) S/m)**
Human Skin (dry)	38	1.46
Human Fat tissue	5.3	0.11
Human Muscle tissue	52.7	1.77
Human Cortical Bone tissue	11.35	0.40

Porcine Skin (dry)	40	1.6
Porcine Fat tissue	10.1	0.29
Porcine Muscle tissue	48.5	2.06
Porcine Cortical Bone tissue	14.8	0.56

**Table 3.** Characteristics.

**Characteristics**	**Subject 1**	**Subject 2**	**Subject 3**	**Subject 4**
Height [cm]	194	186	176	170
Weight [kg]	106	82	76	80
Arm Length [cm]	84	83	75	75
Leg Length [cm]	46	45	44	41
Waist circumference [cm]	106	83	91	103
Chest circumference [cm]	109	99	102	106
Arm circumference [cm]	34	34	28	31
Knee circumference [cm]	43	42	41	38
Thigh circumference [cm]	58	48	53	47

**Table 4.** PER/RSSI at −7 dBm.

**Link/Pos**	**Subject 1**		**Subject 2**		**Subject 3**		**Subject 4**	

	**RSSI**	**PER**	**RSSI**	**PER**	**RSSI**	**PER**	**RSSI**	**PER**
L1/P1	−84	0%	−72	0%	−85	0%	−82	0%
L1/P2	−76	0%	−68	0%	−71	0%	−76	0%
L1/P3	−85	0%	−74	0%	−71	0%	−80	0%
L1/P4	−90	52%	−84	6%	−85	3%	−81	0%
L1/P5	−90	29%	−89	20%	−80	0%	−80	0%

L2/P1	−71	0%	−68	0%	−64	0%	−62	0%
L2/P2	−77	0%	−68	0%	−64	0%	−62	0%
L2/P3	−85	10%	−85	1.4%	−76	0%	−86	7%
L2/P4	−78	0%	−86	1.9%	−65	0%	−65	0%
